# An Allosteric Inhibitory Site Conserved in the Ectodomain of P2X Receptor Channels

**DOI:** 10.3389/fncel.2019.00121

**Published:** 2019-04-09

**Authors:** Ariel R. Ase, Éric Therrien, Philippe Séguéla

**Affiliations:** ^1^Alan Edwards Centre for Research on Pain, Department of Neurology and Neurosurgery, Montreal Neurological Institute, McGill University, Montreal, QC, Canada; ^2^Molecular Forecaster Inc., Montreal, QC, Canada

**Keywords:** purinoceptor, ATP, nucleotides, ionotropic, calcium, inflammation, pain

## Abstract

P2X receptors constitute a gene family of cation channels gated by extracellular ATP. They mediate fast ionotropic purinergic signaling in neurons and non-excitable cell types in vertebrates. The highly calcium-permeable P2X4 subtype has been shown to play a significant role in cardiovascular physiology, inflammatory responses and neuro-immune communication. We previously reported the discovery of a P2X4-selective antagonist, the small organic compound BX430, with submicromolar potency for human P2X4 receptors and marked species-dependence (Ase et al., 2015). The present study investigates the molecular basis of P2X4 inhibition by the non-competitive blocker BX430 using a structural and functional approach relying on mutagenesis and electrophysiology. We provide evidence for the critical contribution of a single hydrophobic residue located in the ectodomain of P2X4 channel subunits, Ile312 in human P2X4, which determines blockade by BX430. We also show that the nature of this extracellular residue in various vertebrate P2X4 orthologs underlies their specific sensitivity or resistance to the inhibitory effects of BX430. Taking advantage of high-resolution crystallographic data available on zebrafish P2X4, we used molecular dynamics simulation to model the docking of BX430 on an allosteric binding site around Ile315 (zebrafish numbering) in the ectodomain of P2X4. We also observed that the only substitution I312D (human numbering) that renders P2X4 silent by itself has also a profound silencing effect on all other P2X subtypes tested when introduced at homologous positions. The generic impact of this aspartate mutation on P2X function indicates that the pre-TM2 subregion involved is conserved functionally and defines a novel allosteric inhibitory site present in all P2X receptor channels. This conserved structure-channel activity relationship might be exploited for the rational design of potent P2X subtype-selective antagonists of therapeutic value.

## Introduction

P2X receptors are ATP-gated nonselective cation channels, formed by the trimeric assembly of homologous subunits encoded by seven genes in mammals (P2rx1–7), resulting in homomeric or heteromeric channels (North, 2002; Saul et al., 2013). P2X channel subunits share a common topology, with intracellular amino- and carboxy-termini, two transmembrane domains and a large ectodomain constrained in a complex folded conformation by five disulfide bridges (Stojilkovic et al., 2005; Hattori and Gouaux, 2012). The P2X ectodomain contains the binding sites for the endogenous agonist ATP and for antagonists, protons and modulatory metallic ions, whereas the transmembrane helical domains form a non-selective cationic pore permeable to calcium ions (Coddou et al., 2011; Habermacher et al., 2016).

Among the mammalian P2X subtypes, the P2X4 receptor is an ATP-gated channel highly permeable to calcium ions that plays a significant pathophysiological role in cardiovascular, inflammatory and neuropathic disorders (Burnstock and Kennedy, 2011). Knockout mice lacking P2X4 receptors display deficits in shear stress-induced vasodilation, indicating a significant contribution of these channels to the regulation of vascular tone by endothelial cells (Yamamoto et al., 2006). P2X4 is also expressed in monocytes, macrophages and microglia where it is involved in the release of inflammatory mediators and cytokines (Bowler et al., 2003; Tsuda et al., 2003; Raouf et al., 2007; Ulmann et al., 2008). In the central nervous system, P2X4 is upregulated at the surface of spinal microglia following peripheral nerve injury and P2X4-dependent BDNF release was shown to trigger the development of neuronal hyperexcitability underlying tactile allodynia in conditions of chronic neuropathic pain (Trang et al., 2012).

Recently, using a stable cell line expressing human P2X4 and high throughput screening with calcium uptake readout, we have reported the identification of the phenylurea BX430 endowed with the properties of a potent non-competitive antagonist with remarkable selectivity for the P2X4 subtype (Ase et al., 2015). Here, we have extended this previous study to identify the domain(s) responsible for the non-competitive inhibitory effect of BX430 on human P2X4 receptor channels. Based on our observation of the differential blockade of human and rodent P2X4 orthologs by BX430 (Ase et al., 2015) and taking advantage of the high conservation of P2X4 primary sequences among vertebrates, we successfully identified a single key extracellular amino acid critical for the non-competitive binding of BX430 on specific P2X4 orthologs. Based on high-resolution structures of invertebrate and vertebrate P2X receptors in apo as well as liganded states (Pasqualetto et al., 2018), we modeled the 3D structure of zebrafish and human P2X4 channels liganded with BX430. Interestingly, the basic structure and function of this BX430-binding domain present in P2X4 is conserved in the P2X gene family and we show that all the P2X subtypes tested share this allosteric inhibitory site.

## Materials and Methods

### Cell Culture and Plasmids

Human embryonic kidney (HEK293) cells were cultured in Dulbecco’s modified Eagle’s medium and 10% heat-inactivated fetal bovine serum (Invitrogen, Carlsbad, CA, USA) containing penicillin and streptomycin. HEK293 cells stably expressing human His-tagged P2X4 channels (hP2X4-HEK293 cells) were a kind gift from R. Alan North (University of Manchester, UK). They were kept in DMEM:F12 (1:1) containing 10% FBS, penicillin and streptomycin, supplemented with G418 (250 μg/ml) for selection. For experiments involving comparisons between wild-type and mutant P2X4 receptors, HEK293 cells were transiently co-transfected with the fluorescent reporter mCherry and the following cDNAs subcloned in pCDNA3 expression vector (DNA ratio 1:5) : wild-type human P2X4 (hP2X4), mutants hP2X4 I312T, I312G, I312D, I312E, I315K, I312A, I312V, I312L, I312F, I312Y, I312W, wild-type rat P2X4, mutant rat P2X4 T312I, bovine P2X4, xenopus P2X4, wild-type zebrafish P2X4 (zfP2X4), mutants zfP2X4 I315T, I315D, ΔzfP2X4(A)-GFP, human P2X1, human P2X1 F308D, human P2X2, human P2X2 I307D, human P2X3, human P2X3 L298D, human P2X5, human P2X5 M313D, human P2X7 or human P2X7 I310D. Site-directed mutations were introduced using the QuikChange method (Agilent). Transfected cells were used for electrophysiological recordings 48 h post-transfection.

### Electrophysiology

Whole-cell patch-clamp recording of hP2X4-HEK293 cells and transiently transfected HEK293 cells (*V*_hold_= −60 mV) were performed using pipettes filled with internal solution, pH 7.2, containing (in mM): 120 K-gluconate, 1 MgCl_2_, 5 EGTA and 10 HEPES. The recording solution, pH 7.4, comprised (in mM): 140 NaCl, 5 KCl, 2 CaCl_2_, 2 MgCl_2_, 10 HEPES and 10 glucose. Membrane currents were recorded using an Axopatch 200B amplifier and digitized at 500 Hz with a Digidata 1330 interface (Axon Instruments, Molecular Devices, Sunnyvale, CA, USA). Only recordings with series resistance below 10 MΩ and stable for the duration of the recording were considered for analysis. The liquid junction potential was calculated to be 3.7 mV and was not compensated. Drugs were dissolved in recording solution and applied using a SF-77B fast perfusion system (Warner Instruments, Hamden, CT, USA) at a rate of 1 ml/min. All experiments were performed at 22°C. For each individual experiment, current amplitudes before and after drug treatment were compared and expressed as a percentage.

### Homology Modeling and Ligand Docking

Putative binding sites were identified using the *Roll* algorithm^1^. Molecular dynamics was performed on the closed state of the P2X4 receptor (pdb 4DW0) using the GROMACS package^1^. The protein was prepared for docking using PREPARE (hydrogen atoms were added, rotamers and tautomers were evaluated). The ligand BX430 was converted from 2D in 3D and hydrogens were added using the CONVERT program. BX430 was prepared for docking using the SMART program. The ligand was docked using the FITTED docking program with the default settings. PREPARE, CONVERT, PROCESS, SMART, and FITTED are part of the FORECASTER platform (Molecular Forecaster, Montreal, QC, Canada). PyMOL is a graphical program distributed as open-source from Schrödinger (Cambridge, MA, USA). Discovery Studio Visualizer is a graphical program from Accelrys (San Diego, CA, USA).

### Statistics

Current amplitudes recorded during ATP applications were measured and mean values were calculated for comparative analysis. Data are presented as mean ± standard error of the mean (SEM) unless indicated otherwise, analyzed using Student’s *t* test, non-paired two-tailed distribution, paired or one-way analysis of variance (ANOVA) followed by a Sidak’s multiple comparisons test.

## Results

### Species-Selectivity of the Antagonist BX430

**Figure 1:**
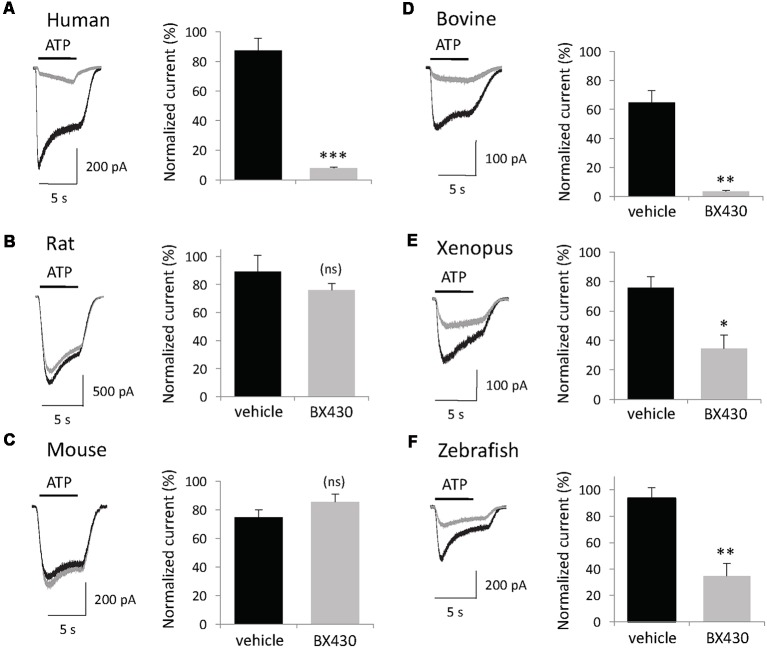
The inhibitory effect of the P2X4 antagonist BX430 compound is species-dependent. **(A,D,E,F)** Representative responses and quantitative results showing ATP (50 μM)-evoked currents recorded in patch clamp from HEK293 cells transfected with human, bovine, xenopus and zebrafish P2X4 receptor channels displaying sensitivity to BX430. **(B,C)** Representative responses and quantitative data showing that rat and mouse P2X4 receptor channels are not affected by treatment with BX430 (10 μM). In all panels: current traces from vehicle-treated cells in black, from BX430-treated cells in gray. *P < 0.05; **P < 0.01; ***P < 0.001 (*n* = 5–7).

The primary sequence of P2X4 subunits is significantly conserved between vertebrates from fish to primates, therefore, we investigated the sensitivity of diverse P2X4 orthologs to the inhibitory effect of BX430, a small organic compound (MW = 413) that blocks selectively human P2X4 channels with submicromolar potency (Ase et al., 2015). Plasmids encoding P2X4 subunits from human, rat, mouse, bovine, xenopus and zebrafish were transiently transfected in HEK293 cells and patch-clamp recording was performed 48 h later. To monitor both desensitization and recovery kinetics, the protocol consisted of several short (5 s) applications of ATP 2 min apart under voltage clamp conditions (Vh = −60 mV). Following the second control application of ATP, transfected cells were exposed to vehicle (DMSO 0.1%) or BX430 for 2 min and then tested for ATP + BX430 (co-application then ATP alone was applied again to measure the recovery response. As shown in Figure 1, BX430 blockade of ATP-evoked current was species-dependent. In spite of high similarity with human P2X4 sequence, rat and mouse P2X4 channels (both 87% amino acid identity) mediated ATP-evoked current responses that were not significantly affected by the application of 10 μM BX430. This lack of sensitivity was also confirmed using higher concentrations of BX430 (up to 100 μM; data not shown). In contrast, bovine P2X4 receptors displayed high sensitivity to BX430 (95% blockade), similar to human P2X4 (91%) while zebrafish and xenopus P2X4 orthologs displayed lower but nevertheless significant sensitivity to blockade by BX430 (63% and 55% inhibition, respectively).

**Figure 2:**
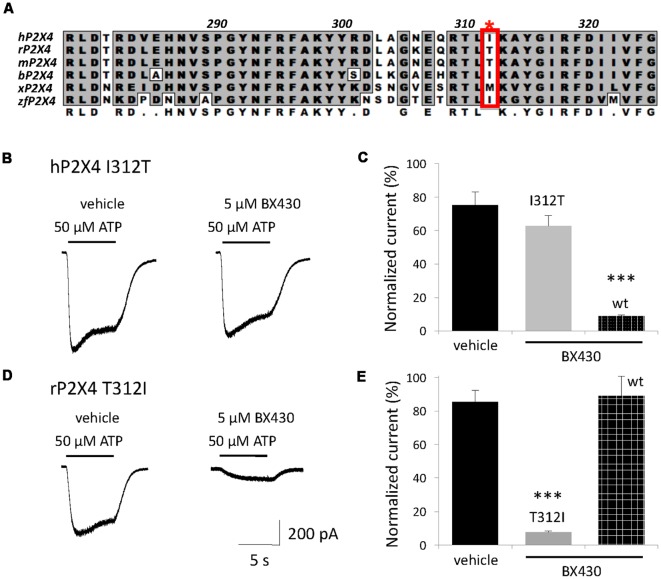
A single residue in the ectodomain, Ile312 in human P2X4, determines BX430 antagonist effect and sensitivity of P2X4 orthologs. **(A)** Alignment of a subregion of the ectodomain of human P2X4 with several vertebrate P2X4 orthologs showing Ile312 in its proximal environment. r, rat; m, mouse; b, bovine; x, xenopus; zf, zebrafish. **(B,C)** Representative traces and quantitative results showing a complete loss of sensitivity to BX430 caused by the single mutation I312T on human P2X4 receptor channels expressed in HEK293 cells. **(D,E)** Representative traces and quantitative results showing the gain of sensitivity to BX430 caused by the single mutation T312I on rat P2X4 receptor channels. ***P < 0.001 (*n* = 5–8).

We previously reported electrophysiological evidence for an extracellular site of action of the ligand BX430 on P2X4 receptors (Ase et al., 2015). The alignment of the ectodomains of P2X4 orthologs (Figure 2A) combined with their differential sensitivity to blockade by BX430 was used as a strategy for the identification of amino acids or subdomains responsible for the inhibitory effect of BX430. We specifically looked for residues identical or similar in the ectodomain of BX430-sensitive human, bovine, xenopus and zebrafish P2X4 orthologs while physicochemically different in BX430-resistant rodent orthologs. Among six candidates, the aliphatic isoleucine Ile312 (human P2X4 numbering) was particularly interesting because it is replaced by polar threonines in BX430-resistant rat and mouse P2X4 subunits. We generated the mutant hP2X4 I312T and observed that this single mutation resulted in an almost complete loss of sensitivity to BX430 (Figures 2B,C). Reciprocally, substituting threonine 312 for isoleucine in the rat sequence (mutant rP2X4 T312I) conferred *de novo* sensitivity to BX430 to the rat P2X4 receptor (Figures 2D,E), demonstrating that the extracellular residue Ile312 in human P2X4 is a necessary component of the binding domain for the antagonist BX430.

### Structural Basis for the Pharmacological Properties of BX430

**Figure 3:**
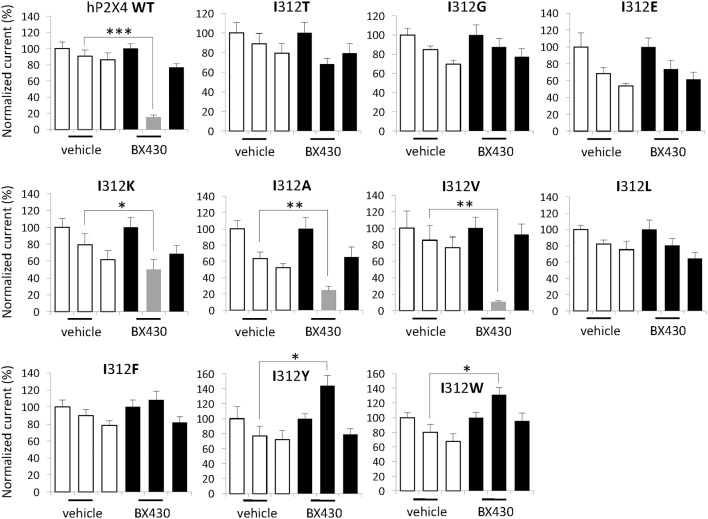
Differential impact of amino acid substitutions at position 312 on the sensitivity of human P2X4 to BX430. Quantitative analysis of the electrophysiological recordings shows that the mutations on human P2X4 I312K, I312A and I312V do not interfere with BX430 binding and blockade, whereas the mutations I312T, I312G, I312E, I312L and I312F induce a loss of sensitivity to BX430. Human P2X4 mutants with I312Y or I312W display significant BX430-evoked potentiation of ATP responses. *P < 0.05; **P < 0.01; ***P < 0.001 (*n* = 6–12).

To look closer into the nature of interactions between BX430 and human P2X4, we carried out a series of systematic substitutions of I312 for amino acids with different side chains, such as polar uncharged, negatively- or positively-charged, aliphatic or aromatic (Figure 3, see also summary of results in Table 1). Except for the only silent mutant hP2X4 I312D, all the mutants of human P2X4 generated responses evoked by 50 μM ATP, with current phenotypes similar to wild-type hP2X4 (data not shown). We observed a similar impact of replacing isoleucine with polar (threonine) or small apolar (glycine) uncharged amino acid, both inducing a loss of sensitivity to BX430 in the mutants I312T and I312G. Substitutions for aspartate and glutamate with negatively charged side chains produced contrasting results: the mutant I312D was silent, even when stimulated by up to 500 μM ATP (data not shown), while the mutant I312E was fully functional but did not display any sensitivity to BX430. The mutation with a positively charged amino acid I312K preserved both functionality and blockade by BX430, but with lower sensitivity. The aliphatic side chain of valine is similar in size to isoleucine and the mutant I312V mirrored the wild-type phenotype regarding sensitivity to BX430. Mutant hP2X4 I312A was also found significantly sensitive to BX430 but with lower potency than the wild-type version. Unexpectedly, when isoleucine was replaced by its regioisomer leucine in the mutant I312L, human P2X4 lost its sensitivity to BX430. BX430 also lost its antagonist properties on the mutants with aromatic side chains I312F, I312Y and I312W. On the contrary, we recorded a significant potentiation of the current response in the presence of BX430 in the cases of I312Y and I312W (Figure 3).

**Table 1:** Summary of electrophysiological data obtained in single amino acid substitution experiments targeting Ile312 (human, h) or Ile315 (zebrafish, zf) in the ectodomain of P2X4 receptor channels.

Mutant	Function	Blockade by BX430
**Polar uncharged side chain**		
hP2X4 I312T	Yes	No
zfP2X4 I315T	Yes	No
**Negatively-charged side chain**		
hP2X4 I312D	No	n.a.
zfP2X4 I315D	No	n.a.
hP2X4 I312E	Yes	No
**Positively-charged side chain**		
hP2X4 I312K	Yes	Yes
**Apolar aliphatic side chain**		
hP2X4 I312G	Yes	No
hP2X4 I312A	Yes	Yes
hP2X4 I312V	Yes	Yes
hP2X4 I312L	Yes	No
**Aromatic side chain**		
hP2X4 I312F	Yes	No
hP2X4 I312Y	Yes	No (potentiation)
hP2X4 I312W	Yes	No (potentiation)

**Figure 4:**
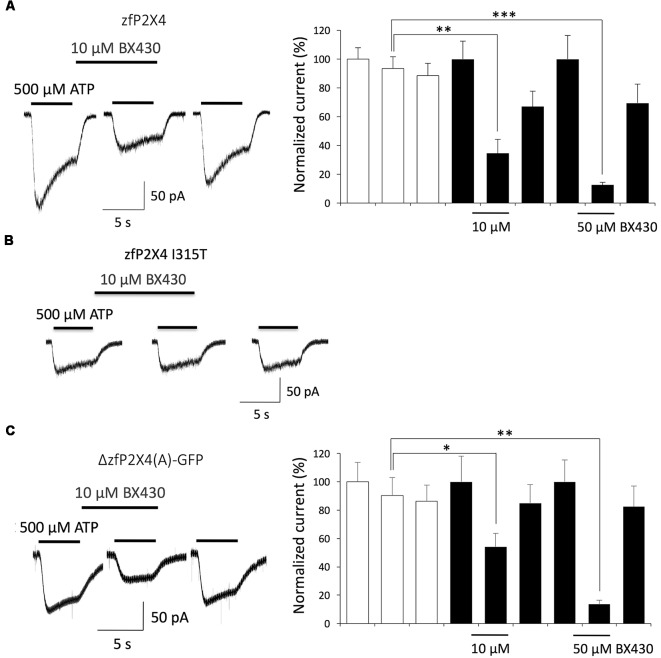
Both wild-type zebrafish P2X4 and the crystallized truncated form ΔzfP2X4(A)-GFP are sensitive to the antagonist BX430. **(A)** Typical ATP-evoked current responses and quantitative results documenting dose-dependent inhibition of wild-type zebrafish P2X4 by BX430. **(B)** Representative traces showing that the mutation I315T in zebrafish P2X4 induces a complete loss of sensitivity to BX430. **(C)** Typical ATP-evoked current responses and quantitative results showing that BX430 is also an antagonist of the truncated form of zebrafish P2X4 ΔzfP2X4(A)-GFP used in X-ray crystallography (Kawate et al., 2009). *P < 0.05; **P < 0.01; ***P < 0.001 (*n* = 5–9).

Reports of the crystal structure of zebrafish P2X4 and other invertebrate and vertebrate P2X subtypes solved at high resolution in closed as well as ATP-bound open states provided a breakthrough in the understanding of structure-activity relationships that underlie gating mechanisms by specific nucleotides, desensitization, antagonist binding or ion selectivity of P2X channels (review in Pasqualetto et al., 2018). We first checked that wild-type zebrafish P2X4 is effectively blocked by BX430 in a dose-dependent manner (Figure 4A) and confirmed that the residue Ile315 homologous to Ile312 in human P2X4 plays also a key role in the inhibitory effect of BX430. Moreover, substituting Ile315 for threonine effectively suppressed blockade of zebrafish P2X4 by BX430 (Figure 4B): as for hP2X4 I312T, zfP2X4 I315T became insensitive to 10 μM BX430 (vehicle: 98 ± 5.5% vs. BX430 95 ± 4.5%; *n* = 5). Compared to wild-type, the mutant zfP2X4 I312T was also found less sensitive to 500 μM ATP (peak current amplitude = 44 ± 10 pA for mutant vs. 147 ± 13 pA for wild-type, *n* = 5). In order to use them as structural references for the 3D modeling of human P2X4, we wondered then if the truncated forms of zebrafish P2X4 that were crystallized were also subject to blockade by BX430. As shown in Figure 4C, the truncated mutant ΔzfP2X4(A)-GFP (Kawate et al., 2009) expressed in HEK293 cells was significantly inhibited by BX430, with 56% blockade at 10 μM and 86% blockade at 50 μM. Thus, both the wild-type and its truncated counterpart show similar sensitivity to the antagonist BX430. We were not able to reach sufficient levels of surface expression in HEK293 cells for the other reported versions of crystallized truncated zfP2X4 constructs in HEK293 cells or the mutant ΔzfP2X4(A)-GFP I315T (data not shown). Nevertheless, we could conclude that human and zebrafish P2X4 receptors share homologous binding sites for the antagonist BX430 around the same Ile312/315 residue in their ectodomain.

**Figure 5:**
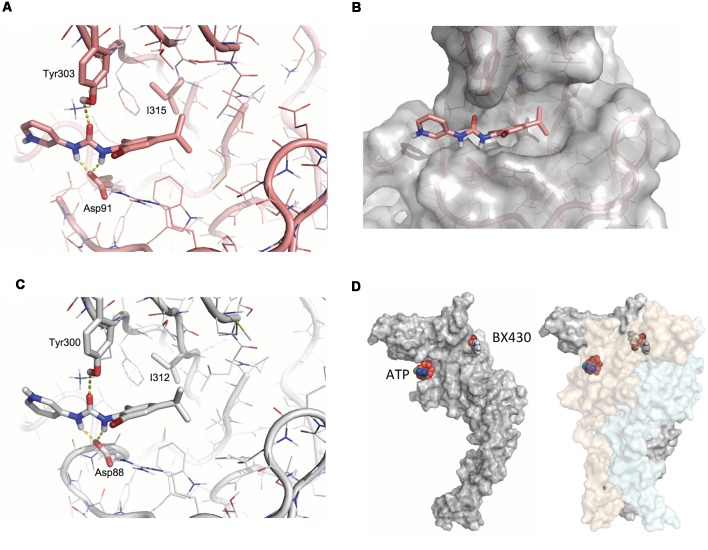
Modeling of the BX430 binding domain in zebrafish and human P2X4 structures. **(A)** Docking of the antagonist BX430 using molecular dynamics simulation on the model of zebrafish P2X4 based on X-ray crystallographic data of the construct ΔzfP2X4(A)-GFP (pdb code 4DW0). Key isoleucine (Ile315), as well as tyrosine (Tyr303) and aspartate (Asp91) residues contributing to the binding of BX430, are indicated. **(B)** 3D surface representation of docked BX430 in ΔzfP2X4(A)-GFP. **(C)** Homologous docking of BX430 on the model of human P2X4 subunit and key amino acids involved in the binding domain. **(D)** Model of a single human P2X4 subunit in surface view (left) and global quaternary structure of the trimeric P2X4 complex (right), illustrating the relative positions of the agonist-binding site occupied by ATP and the allosteric antagonist-binding site occupied by BX430.

Available crystal structures of P2X4 receptor channels were inspected (pdb 4DW0, 4DW1, 3H9V, and 3I5D) and the structure of zebrafish P2X4 in its closed, apo state (pdb code 4DW0) was selected to identify putative BX430 binding sites by detecting pockets and cavities on the receptor using complementary *Roll* and DogSiteScorer modeling approaches. The docking sites in zebrafish P2X4 were set to sample the region proximal to Ile315 in the upper body region of the channel subunit. The ligand BX430 was prepared using the FORECASTER platform. The docking poses were generated for the identified putative binding sites using the rigid and flexible protein docking modes available in the FITTED program. Visual inspection of the proposed poses led to the identification of the binding site that showed the best interactions with most of the relevant residues (Figures 5A,B).

Homology models for human P2X4 were built based on the zebrafish P2X4 crystal structure (pdb code 4DW0) using the SWISS-MODEL web server. Docking poses were generated by the same protocol using the FITTED program. Visual inspection of the proposed poses led to the selection of the best receptor conformations—binding sites combination. The selected docking modes of BX430 for human P2X4 were used as starting structures for molecular dynamics simulations. The simulations were performed in GROMACS (version 4.5.4) with the amber ff99sb and GAFF force fields using AM1-BCC partial charges for BX430. The receptor and BX430 were solvated in a triclinic TIP3P water box and neutralized by adding counter-ions. The systems were energy-minimized to ensure the systems have no steric clashes or inappropriate geometry. The energy-minimized systems were equilibrated with position restraints for 100 ps at constant volume and temperature (300 K). Production runs were for 2 ns with 2-fs time steps using the LINCS algorithm to constrain bonds between hydrogens and heavy atoms. Trajectories were analyzed using PyMOL. Representative conformations were extracted from clustering and up to three structures were selected for further evaluation. Then, the BX430 ligand was docked within these conformations using the flexible protein docking mode of FITTED. Using the human P2X4 model as a template, homology models for the mutated hP2X4 I312T structure was built. The BX430 ligand was docked to each model and the region around Ile/Thr312 in the upper body region was used to define the overall docking site of BX430 on wild-type and mutant human P2X4 receptor (Figure 5C). The identification of negative charges (Asp88) and hydrogen bond donor (Tyr300) around the Ile312 residue was the starting point for molecular docking studies. We propose that the amines in the urea moiety of BX430 interact with the C = O of Asp88 while the C = O of BX430 makes an H-bond with the hydroxyl of Tyr300. The dibromo-isopropylphenyl group of BX430 fills a hydrophobic pocket around Ile312 and Leu107 (Ile110 in zebrafish P2X4; Figures 5A–C) that is disrupted in the mutant Ile312T while the pyridine moiety of BX430 is solvent-exposed and interacts with surrounding water molecules. The significant physical distance observed between the docking site of BX430 and the ATP-binding site (Figure 5D) provides a structural basis for the non-competitive nature of the inhibition induced by the binding of BX430 on P2X4 receptor channels.

### The Function of the BX430-Binding Domain in P2X4 is Conserved in the Whole P2X Family

**Figure 6:**
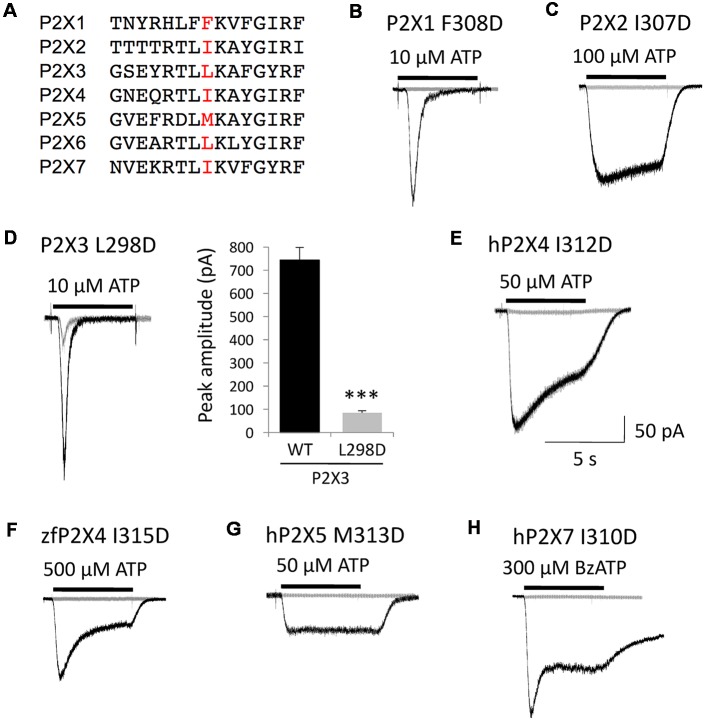
The silencing mutation I312D in human P2X4 disrupts a key functional domain conserved in the P2X gene family. **(A)** Alignment of partial primary sequences of human P2X channel subunits with a focus on the homologous extracellular domain around Ile312 in P2X4. **(B–H)** Representative agonist-evoked current traces showing the profound inhibitory impact of mutation I312D on human P2X4 and of the equivalent substitutions indicated on all other human homomeric P2X subtypes, as well as on zebrafish P2X4. **(D)** Whereas all other subtypes are rendered silent, the P2X3 receptor remains functional although its ATP-gated channel activity is severely disrupted by the mutation L298D, as indicated by quantitative comparison of peak current amplitudes. ***P < 0.001 (*n* = 6–13).

Only one specific mutation in human P2X4 receptor, isoleucine to aspartate (I312D), had a profound functional impact by itself as P2X4 channels became completely insensitive to ATP. This suggested a critical role for this Ile312 residue and/or its immediate surrounding environment in the basic function of P2X4 receptor channels. Taking into account the high level of conservation of this pre-TM2 subdomain in the P2X family (see the alignment in Figure 6A), we considered the possibility that the strong antagonist effect of this single mutation might be extended to other P2X channel subtypes. We mutated all human P2X receptor subunits known to assemble into functional homotrimers (P2X1–4, P2X5–7) by substituting the residue homologous to Ile312 in human P2X4 (either isoleucine, leucine or methionine in the other P2X subtypes) for aspartate. These mutant receptors, i.e., P2X1 I308D, P2X2 I307D, P2X3 L298D, P2X4 I312D, P2X5 M313D and P2X7 I310D, were expressed in HEK293 cells and tested for agonist (ATP or BzATP)-evoked current responses (Figures 6B–H). We did not test mutant human P2X6 because the wild-type homomeric P2X6 channel is insensitive to ATP. As illustrated in Figure 6, all the homomeric P2X subtypes tested were rendered silent by this mutation, except P2X3 L298D. However, the response of the mutant P2X3 channels to ATP was severely impaired, as the amplitude of their peak currents was about 10 times lower compared to wild-type P2X3 (Figure 6D). The homologous mutation introduced in zebrafish P2X4, I315D, fully suppressed channel activity (Figure 6F). These results indicate that this aspartate mutation targeting a conserved subregion of the ectodomain, which happens to be a BX430-binding site in P2X4 selectively, disrupts a basic functional mechanism shared by all the P2X receptor subtypes tested.

## Discussion

Altogether, our results provide convincing evidence for the existence of a novel allosteric binding site for the selective antagonist BX430 in the ectodomain of P2X4 receptor channels. By comparing the primary sequences of several BX430-sensitive and -resistant P2X4 orthologs, we were able to identify one extracellular residue (at position 312 in human P2X4) in the ectodomain of P2X4 as the single critical determinant for the inhibitory effect of BX430. The structure of the subdomain involved is conserved in the whole P2X family and we showed that its disruption has a generic silencing effect on the function of all P2X receptor channels.

### Molecular Basis for Species-Specific Sensitivity to the Antagonist BX430

We have reported that the P2X4-selective antagonist BX430 is an effective blocker of human P2X4 with submicromolar potency but it has no measurable effect on mouse P2X4 receptors (Ase et al., 2015). Previous cases of species-specific pharmacology in the field of P2X receptors include, among others, the P2X3 antagonist RO51 (Serrano et al., 2012), the P2X4 antagonists suramin and PPADS (Garcia-Guzman et al., 1997; Jones et al., 2000), the P2X7 antagonist AZ11645373 (Michel et al., 2009) and the positive allosteric modulation of P2X7 by ivermectin (Nörenberg et al., 2012). We tested several other vertebrate P2X4 orthologs (rodents, bovine, xenopus, zebrafish) for their sensitivity to BX450 and noticed that the antagonist property of BX430 is not directly related to the degree of homology with human P2X4. Human and bovine P2X4 (94% sequence similarity) share a high sensitivity to BX430. However, xenopus and zebrafish P2X4 receptor channels (only 81% and 75% similarity with human P2X4, respectively) also display a significant sensitivity to BX430 while rodent P2X4 orthologs (93% sequence similarity with human P2X4 for rat and mouse subunits) are resistant to BX430 blockade. All vertebrate P2X4 receptors tested display similar current phenotypes, therefore, a differential effect of BX430 independent from the global homology strongly implies that a specific sequence or subdomain is shared between BX430-sensitive P2X4 orthologs. Focusing on the ectodomain, which was found to be site of action of BX430 (Ase et al., 2015), we identified a hydrophobic residue (isoleucine or methionine) at position 312 (human numbering), present in all BX430-sensitive P2X4 receptors but replaced by the polar residue threonine in rodents, as the determinant of BX430 antagonist effect. Indeed we demonstrated that Ile312 plays a critical role by introducing the loss-of-inhibition mutation I312T in human P2X4 and showing that it converts it into a rodent-like BX430-insensitive receptor. Reciprocally, we conferred a BX430-sensitive phenotype to the rat P2X4 receptor channel by introducing the gain-of-inhibition mutation T312I, convincingly proving that the nature of the amino acid at position 312 determines sensitivity or resistance to BX430.

Since isoleucine and methionine are two hydrophobic amino acids with an aliphatic side chain, these physicochemical properties might be required for BX430 binding and inhibitory effects. This was tested by swapping isoleucine for various amino acids. Systematic single mutations on the isoleucine315 of human P2X4 with amino acids with different types of side-chain structures (see Table 1) revealed that its substitution by amino acids with negatively-charged polar, polar uncharged, or aromatic side chains renders the P2X4 channel either silent or insensitive to blockade by BX430. Unexpectedly, replacing isoleucine with its regioisomer leucine or with glycine also disrupts BX430 sensitivity while inserting the bulky aromatic side chains of tyrosine and tryptophan at position 312 in human P2X4 converts BX430 into a positive allosteric modulator. The residues preserving BX430 sensitivity were found to be the hydrophobic alanine or valine and to a lesser extent the positively-charged lysine.

From this series of mutagenesis experiments, apart from the exclusion of polar uncharged, negatively-charged or aromatic side chains at position 312 (in human P2X4), the diversity of side chains compatible with binding and inhibitory effects of BX430 confirms the folding complexity of the ectodomain in P2X channel subunits. We took advantage of the fact that the homologous isoleucine in the ectodomain of wild-type zebrafish P2X4 (Ile315) is also critical for its blockade by BX430, and that the crystallized form ΔzfP2X4(A)-GFP remains sensitive to BX430, to look at the 3D structure of the BX430 binding pocket in models of zebrafish and human P2X4 receptors. Molecular dynamics simulation based on the crystal structure of ΔzfP2X4(A)-GFP allowed us to identify a groove at the surface of the ectodomain around Ile315 where BX430 could bind with relevant affinity. A homologous binding domain was confirmed in the virtual model of human P2X4, also based on ΔzfP2X4(A)-GFP. These simulations predict that Tyr300 and Asp88, with Ile312 (human numbering), participate in the coordination of the BX430 binding. Another interesting observation from these structural models is that the docking site for BX430 was found to not overlap with the ATP binding site, confirming our empiric results on the noncompetitive allosteric nature of the inhibitory effects of BX430 (Ase et al., 2015). From these modeling data, we conclude that the most likely mechanisms of action of BX430 on P2X4 receptor channels is a structural locking of this subregion of the ectodomain (Jiang et al., 2013; Mansoor et al., 2016) induced by its binding to the allosteric site involving Asp88, Tyr300 and Ile312 in human P2X4. Locking or stabilization mechanisms involving different parts of the ectodomain have been proposed for the inhibition of P2X3 by the antagonists TNP-ATP (Mansoor et al., 2016) and AF-219 (Wang et al., 2018), and also for the inhibition of P2X7 by TNP-ATP (Kasuya et al., 2017) based on crystal structures.

### A Key Functional Domain Conserved in the P2X Family

In our extended series of amino acid substitutions targeting Ile312 in human P2X4, the only mutation that induced a loss of ATP-gated channel activity in the absence of antagonist was I312D. It remains to be elucidated why the mutation to glutamate, also with a negatively charged side chain, does not affect hP2X4 as does aspartate. Nevertheless, such a strong functional impact of replacing a single isoleucine residue with aspartate highlighted an important role of Ile312 and its proximal environment in the normal function of the P2X4 channel. The selective binding of BX430 likely requires multiple P2X4-specific interactions beyond the key conserved residues identified in our docking model. However, as all P2X receptor channels share similar basic mechanisms of operation and a significant degree of homology in the corresponding pre-TM2 region, we predicted that mutation of this binding domain in other P2X subtypes would have similar loss-of-function effects. Indeed, virtually all human P2X subtypes, as well as zebrafish P2X4, were fully silenced by a single mutation replacing the residue at position 312 (human P2X4 numbering) with aspartate. The only exception was the mutation L298D introduced in P2X3, although the channel activity was severely reduced. Therefore, we can conclude that this aspartate mutation reveals the existence of a conserved inhibitory allosteric site present in most, if not all, P2X receptor subtypes. Interestingly, the residue I310 in P2X7 (homologous to I312 in P2X4) has been reported to be in close proximity to a non-competitive allosteric antagonist site and the mutation I310C was found to disrupt P2X7 channel activity after modification with the cysteine reagent MTS-TPAE (Karasawa and Kawate, 2016). Altogether, this provides further evidence that the structure around I312 (human P2X4 numbering), located in β-strand #14 in the pre-TM2 region of the “upper body” of P2X subunits (Hattori and Gouaux, 2012), plays a key role in P2X function.

A cluster of positively-charged amino acids is responsible for coordinating the phosphate groups of the bound ATP (Evans, 2010; Hattori and Gouaux, 2012) and one of these, Lys313 (human P2X4 numbering), is adjacent to the aspartate mutation in all the P2X receptors. This might lead to the neutralization of the positive charge of Lys313 and could explain the insensitivity to ATP. Alternatively, the aspartate mutation could compromise the channel by disrupting the proper folding of the ectodomain required for trimeric assembly, for translocation to the plasma membrane and/or for gating the opening of the cationic pore. We did not observe a dominant-negative effect of the mutant P2X4 I312D subunit on the current amplitude of co-expressed wild-type P2X4 channels (data not shown), therefore a disruption of subunit oligomerization and/or trafficking is unlikely. The structural changes induced by the aspartate mutation could mimic the inhibitory effects of BX430 bound on P2X4 receptor channels by hindering ATP-induced gating movements (Jiang et al., 2013; Karasawa and Kawate, 2016; Wang et al., 2018).

From a translational point of view, the localization of a generic allosteric inhibitory site conserved in all P2X receptors might facilitate the discovery and design of P2X subtype-selective non-competitive antagonists of therapeutic value, for example through the virtual screening of compound libraries on high-resolution crystal structures.

## Author Contributions

AA performed the experiments, analyzed the data and wrote the manuscript. ÉT performed modeling, molecular dynamics simulations and wrote the manuscript. PS designed the project, analyzed the data and wrote the manuscript.

## Conflict of Interest Statement

ÉT was employed by the company Molecular Forecaster Inc. The remaining authors declare that the research was conducted in the absence of any commercial or financial relationships that could be construed as a potential conflict of interest.

## Footnotes

^1^http://www.gromacs.org/
